# Multimodality Imaging in Right Heart Tumors: Proposed Algorithm towards an Appropriate Diagnosis

**DOI:** 10.3390/jcm13041000

**Published:** 2024-02-09

**Authors:** Mariana Floria, Alexandru Burlacu, Paula Cristina Morariu, Alexandru-Florinel Oancea, Diana-Elena Iov, Genoveva Livia Baroi, Celina Silvia Stafie, Viorel Scripcariu, Daniela Maria Tănase

**Affiliations:** 1 Department of Internal Medicine, Faculty of Medicine, University of Medicine and Pharmacy Grigore T. Popa, 700115 Iasi, Romania; floria.mariana@umfiasi.ro (M.F.); alexandru.burlacu@umfiasi.ro (A.B.); morariu.paula-cristina@email.umfiasi.ro (P.C.M.); alexandru.oancea@umfiasi.ro (A.-F.O.); daniela.tanase@umfiasi.ro (D.M.T.); 2 Saint Spiridon Emergency Hospital, 700115 Iasi, Romania; celina.stafie@umfiasi.ro; 3 Institute of Cardiovascular Diseases, 700503 Iasi, Romania; 4 Department of Surgery, Faculty of Medicine, University of Medicine and Pharmacy Grigore T. Popa, 700115 Iasi, Romania; viorel.scripcariu@umfiasi.ro; 5 Department of Preventive Medicine and Interdisciplinarity, Faculty of Medicine, University of Medicine and Pharmacy Grigore T. Popa, 700115 Iasi, Romania; 6 Regional Institute of Oncology, 700483 Iasi, Romania

**Keywords:** right heart, tumor, multimodality, imaging, echocardiography, computer tomography, cardiac magnetic resonance, positron emission tomography

## Abstract

A right heart tumor can be identified by transthoracic echocardiography during a routine examination or due to cardiac symptoms. The first step is the assessment by echocardiography, with its multiple techniques, and the obtained information must be judged in a clinical and biological context. The second step comprises one, sometimes even two, of the more complex modality imaging methods. The choice is driven not only by the advantages of each imaging technique but also by local expertise or the preferred imaging modality in the center. This step is followed by staging, follow-up, and/or imaging-guided excision or biopsy, which is performed in selected cases in order to obtain anatomopathological confirmation. In the presence of features suggestive of malignancy or causing hemodynamic impairment, a transvenous biopsy is essential before the more complex imaging modalities (which are still relevant in the staging process). Using a structured imaging approach, it is possible to reach an appropriate diagnosis without a biopsy. Frequently, these imaging techniques have a complementary role, so an integrated imaging approach is recommended. This proposed algorithm for appropriate diagnosis of right heart tumors could serve as a practical guide for clinicians (not only imaging specialists).

## 1. Introduction

Numerous masses can be identified within the chambers of the right heart and may be evaluated using various imaging modalities, including echocardiography, computer tomography (CT), positron emission tomography–CT (PET–CT), cardiac magnetic resonance (CMR) imaging, scintigraphy, or even invasive angiography [1,2,3,4,5]. These structures can be composed of normal bands, aberrant structures, or other lesions. As they can mimic cardiac tumors, these masses often pose a diagnostic challenge. The normal variants typically appear in a characteristic location and exhibit the same signal intensity and enhancement characteristics as the normal myocardium. By contrast, right heart tumors have altered signal intensity and enhancement properties, which entail the use of several imaging methods followed by excision or biopsy [4]. These tumors can be primary (benign or malignant) or secondary (metastatic).

Primary malignant heart tumors are exceptionally rare, comprising less than 0.2% of cardiac tumors [4]. Therefore, fewer data are available about these primary malignant right heart tumors. In adults, around 75% of primary cardiac tumors are benign, a percentage that increases up to 90% in children [6]. It appears that about 80% of all benign primary cardiac tumors are rhabdomyomas and fibromas [7]. Breast cancer, lymphoma, and melanoma are the most common metastatic cancers affecting the heart [5]. Benign tumors may have a malignant counterpart [8], such as myxoma (myxosarcoma), lipoma (liposarcoma), rhabdomyoma (rhabdomyosarcoma), angioma (angiosarcoma), fibroma (fibrosarcoma), schwannoma (malignant peripheral nerve sheath tumor), etc. For papillary fibroelastoma, the corresponding primary malignant tumor could be a lymphoma or mesothelioma. Cardiac lymphomas grow at a very fast rate and can be identified either in the right atrium, right ventricle, or pericardial space [8].

It seems that cardiac metastases often involve the right side of the heart.

Right heart tumors are usually identified by transthoracic echocardiography (TTE) during a routine examination or due to cardiac symptoms [8]. Given their ability to mimic various cardiac pathologies, cardiac tumors may present with diverse clinical features. These clinical presentations are taken into consideration when a right heart tumor is detected on TTE or transesophageal echocardiography (TEE) examination.

It is crucial to recognize individual clinical contexts and settings, encompassing factors like medical history, age, gender, ethnicity/geography, and risk factors, before selecting an imaging method [4,9]. Significant features such as fever, weight loss, fatigue, or paraneoplastic syndromes, along with phenomena like embolism, cardiac dysfunction resulting from arrhythmias or valvular insufficiency, and hemodynamic compromise, play a pivotal role. Some tumors occur in younger patients, whereas others occur in older individuals [9]. Risk factors such as intravenous drug use, recent invasive medical procedures, impaired systolic function, atrial fibrillation, or a history of thrombotic disease are important when deciding on an appropriate imaging modality or if imaging findings are inconclusive.

Right heart tumors might involve not only the walls (myocardium, endocardium, and pericardium) but also the valves and cardiac chambers [4]. A cardiac mass could be visible on the endocardium but could also invade the myocardium (or pericardium).

Imaging methods often play a complementary role; therefore, an integrated imaging approach is recommended. For example, CT and CMR provide high-resolution anatomic information, while PET–CT can distinguish between benign and malignant tumors by assessing the metabolic activity of tumors (thus providing functional information). The multimodal imaging approach for right heart tumors is the first step towards an appropriate diagnosis, followed by staging, follow-up, and/or imaging-guided excision. Anatomopathological diagnosis must always be confirmed by endomyocardial or excisional biopsy.

However, prior to biopsy or excision, the multimodal imaging examination has an important role in the differential diagnosis of cardiac tumors. Unfortunately, there is a lack of standardization of these imaging modalities with regard to an algorithm, as this is dependent on the locally available expertise of each center or the preferred imaging modality. Therefore, this topic deserves a thorough discussion.

## 2. Imaging Modalities for Assessing Right Heart Tumors

Cardiac imaging has undergone significant advancements over the last decades. Multimodality imaging facilitates the differential diagnosis of right cardiac tumors [4]. Some authors consider that, when available, all imaging methods should be used, especially in certain cardiac tumors like myxofibrosarcoma [7,10].

Regardless of the type of cardiac tumor, an ideal multimodality imaging algorithm has the following objectives [4]:

1. Identify, localize, and describe the tumor (including its origin, attachments, size, morphology, mobility, hemodynamic impact, tissue characteristics, and vascularity);
2. Differentiate the tumor from other cardiac pathologies through a comprehensive differential diagnosis;
3. Assess the consequences of the tumor;
4. Evaluate whether the tumor is primary or secondary.

The utility of the main imaging methods, through the objectives mentioned above, in right heart tumors is detailed in Table 1.

### 2.1. Chest Radiography

Chest radiography is no longer recommended in the evaluation of right heart tumors but is important in the differential diagnosis of murmurs, dyspnea, or signs of right heart failure. In patients with these complaints, it could reveal changes such as cardiomegaly, enlargement of the right chambers, or a dilated pulmonary artery. Depending on the presentation of right heart tumors, a chest X-ray can therefore be part of the imaging evaluation of these patients.

### 2.2. Echocardiography

Two-dimensional transthoracic echocardiography is the first-line non-invasive imaging modality used in the evaluation of heart tumors. It detects cardiac tumors with 90% sensitivity and 95% specificity [7,11].

**Figure 1:**
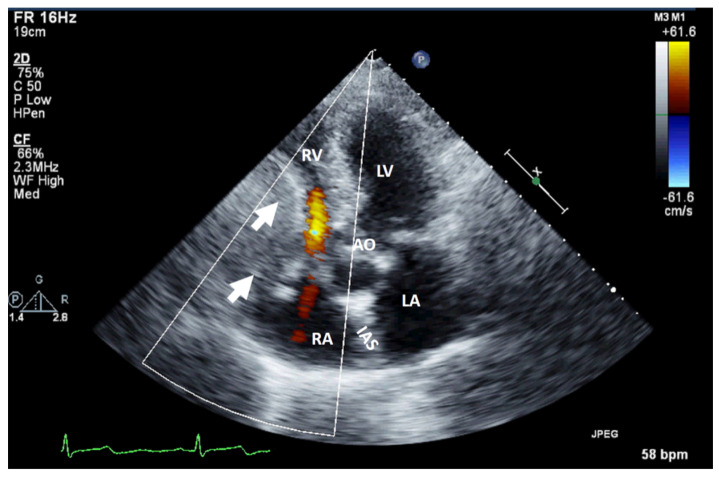
Two-dimensional transthoracic echocardiography (apical 4-chamber view) with color Doppler showing external compression of the free right ventricular wall (arrow) in a patient with hepatomegaly due to a hepatic tumor, which explains hemodynamic instability (similar to a localized cardiac tamponade). AO, aorta; IAS, interatrial septum; LA, left atrium; LV, left ventricle; RA, right atrium; RV, right ventricle.

It can be used as a screening imaging method, but right heart tumors could be an incidental finding during a routine echocardiographic examination. This imaging technique is widely available (accessible), portable, and easy to perform, even at the patient’s bedside and in those who are hemodynamically unstable. It is safe, repeatable, cost-effective, and has a good spatial resolution (higher with TEE) and an excellent temporal resolution. For imaging small, highly mobile masses (<1 cm) or masses arising from valves, it is the optimal imaging modality [4]. TEE is more sensitive at identifying tumors smaller than 5 mm compared with TTE. Therefore, compared with TTE, TEE is better for the detection, location, and mobility of cardiac tumors and equivalent in the assessment of hemodynamic impact, compromising the compression/destruction/distortion of cardiac structures [12]. In some cases, the mass effect of a non-cardiac tumor severely compromises cardiac hemodynamics (Figure 1).

Rhabdomyomas (Video S1, Supplementary Materials) and fibromas occur in younger patients, whereas papillary fibroelastoma tends to occur in older individuals.

**Figure 2:**
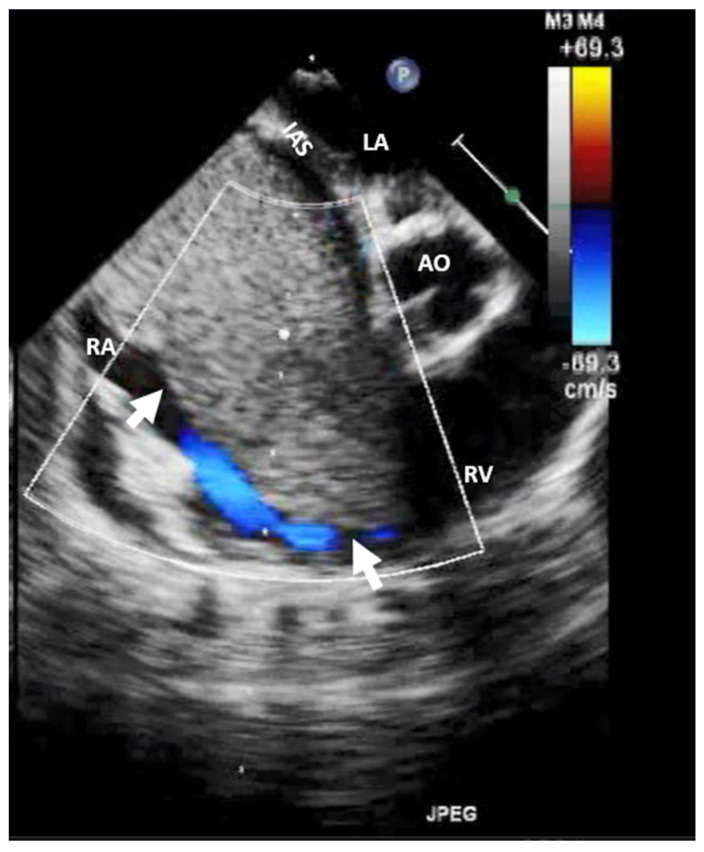
Two−dimensional transesophageal echocardiography (short-axis view at the level of the great vessels) showing a huge inhomogeneous mass (arrow) attached to the interatrial septum crossing the tricuspid valve (which, upon histology diagnosis, was a myxoma). AO, aorta; IAS, interatrial septum; LA, left atrium; RA, right atrium; RV, right ventricle.

Echocardiography with ultrasound-enhancing agents is the best modality for tissue characterization [13]. Two-dimensional TTE may sometimes overrate the size of myxomas or very soft tumors (myxoid- or gelatinous-type masses) due to their distensibility, mobility, or irregular shape, especially when compared to three-dimensional TEE. However, the size of the tumor is appropriately assessed by TTE in most cases. The site and type of tumor attachment, a useful clue to define the type of cardiac tumor, could be determined easily by TEE (especially 3D). For example, right atrium angiosarcomas, described as heterogenic masses with central necrosis on CT or CMR [13], could be attached to any part of the right atrial wall, while myxomas (Figure 2) are usually situated in the fossa ovalis region of the atrial septum (most frequently on the left side).

Echocardiography is highly operator-dependent, has a limited acoustic window in some patients, a narrow field of view, and limited tissue characterization. In addition, TEE requires sedation and necessitates precautions in cases of severe esophageal disease, but it allows a better assessment of the right heart cavities compared to TTE.

All echocardiographic modalities, including two- or three-dimensional color Doppler imaging, spectral Doppler imaging, the use of agitated saline (Video S2—Supplementary Materials), as well as ultrasound enhancement agents, are important in the diagnosis [14] and even in guiding the biopsy procedure for histological examination. Besides guiding potential therapeutic interventions, echocardiography is important for defining the morphology, assessing the size, observing the dynamic appearance, and evaluating functional abnormalities and hemodynamic consequences before other imaging modalities.

**Figure 3:**
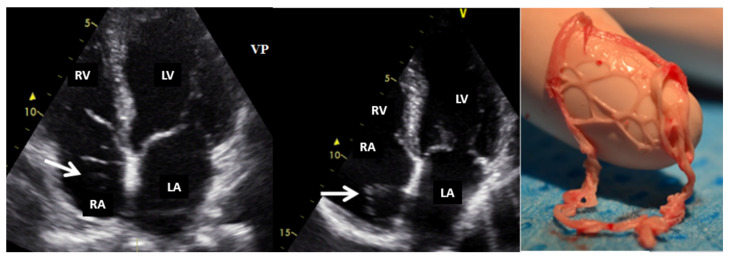
Two-dimensional transthoracic echocardiography (apical 4-chamber view) showing a Chiari network (arrow) in the right atrium before and after (the last image) an electrophysiological study complicated by catheter entrapping in the Chiari apparatus. LA, left atrium; LV, left ventricle; RA, right atrium; RV, right ventricle.

**Figure 4:**
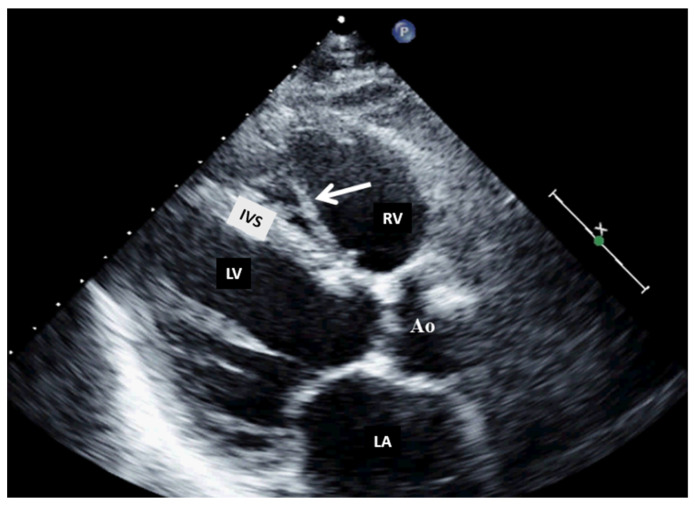
Two-dimensional transthoracic echocardiography (modified parasternal view, long axis) showing a moderator band in the right ventricle. Ao, aorta; IVS, interventricular septum; LA, left atrium; LV, left ventricle; RV, right ventricle.

Normal or aberrant structures that may be present in the right cavities include the crista terminalis, a Chiari network (Figure 3) [15], aberrant atrial bands in the right atrium, a moderator band (Figure 4), aberrant papillary muscles, and accessory chordae tendineae in the right ventricle.

Regarding the clinical presentation and echocardiographic characteristics of the masses [8] identified at the level of the right atrium or ventricle, it is essential to know that:

- Angiosarcomas and lymphomas are the most frequent types of primary malignant tumors of the right heart chambers;
- Liver or ovarian cancers are the most frequent malignant tumors that metastasize to the right heart;
- Angiosarcomas or lymphomas are more likely to infiltrate the atrial wall or extend to the pericardium;
- If there is continuity with the inferior vena cava, consider assessing for liver or gynecological cancers; also, a thorough differential diagnosis with a thrombus or tumor thrombosis should be performed;
- If there is continuity with the superior vena cava, consider lymphoma as a possible diagnosis;
- In the presence of a single mass with a thin stalk, consider myxoma as a possible benign tumor; sessile masses are most likely infiltrative and malignant.

Any right heart tumor with intramural or intrapericardial extension identified by CT, CMR, or PET scan is most probably a malignant tumor.

### 2.3. Cardiac Computer Tomography

Computer tomography is widely available nowadays, has a rapid turnaround time, a high isotropic spatial resolution (better than temporal resolution), superior anatomic depiction, a wide field of view, and multiplane reconstruction possibilities. The main strengths of cardiac CT include the assessment of intrathoracic anatomy, the coronary tree, and the staging of malignant tumors [9]. Unfortunately, this examination requires expertise, exposes individuals to radiation, involves the use of potential nephrotoxic iodinated contrast material, and is challenging to perform in patients who are hemodynamically unstable (as the sequences are breath-hold dependent) or in those with severe arrhythmias [9].

Similar to echocardiography, right heart tumors might be an incidental finding during the CT examination. After echocardiography, cardiac CT or CMR imaging are second-line approaches for assessing right heart tumors. In clinical settings (medical history, age, gender, ethnicity/geography, and clinical risk factors), CT imaging can characterize tumor morphology, location, and surrounding structural assessment. In addition to that, it allows for a better assessment of the morphology and function of the right cavities compared to echocardiography.

Using multidetector cardiac CT in right heart tumors, the following imaging features could be obtained [16]:

1. Size of the mass (assessed in terms of diameters or volumes);
2. Tumor margins (circumscribed, micro-lobulated, obscured or partially hidden by adjacent tissue, indistinct or ill-defined, and spiculated);
3. Invasiveness (disruption of neighboring tissue and extension of the mass into the tissue);
4. Mass density, defined as solid or mainly cystic and rated as hypo-, iso-, or hyperdense (compared to the normal cardiac muscle);
5. Presence of intramass calcifications;
6. Contrast uptake, defined as an increase of at least 10 Hounsfield units in cardiac mass density compared to baseline;
7. Pericardial effusion.

In order to better define surgical approaches for cardiac tumors that might directly involve or abut coronary arteries, cardiac CT could be more useful than CMR. Moreover, cardiac CT offers an alternative to CMR in many patients because of its high isotropic spatial and temporal resolution, multiplane image-reconstruction capabilities, and fast acquisition time [4].

Cardiac CT may be useful in differentiating between benign and malignant cardiac tumors based on irregular borders, invasive appearance, calcification, and Hounsfield units. However, one should bear in mind the fact that imaging appearance may not always be predictive of malignancy. Furthermore, Hounsfield unit values overlap when differentiating hypoperfused myocardium from thrombus.

### 2.4. Cardiac Magnetic Resonance Imaging

If echocardiography serves as the initial step in cardiac tumor assessment, CMR is the preferred imaging modality following echocardiography. This preference stems from its ability to offer a comprehensive, non-invasive evaluation using techniques such as late gadolinium enhancement, post-contrast long-inversion-time imaging, first-pass perfusion, and T1/T2 tissue characterization. This imaging method is most useful for tissue characterization and hemodynamics. CMR is not indicated for the evaluation of valvular vegetations compared to echocardiography because of its lower temporal resolution [4]. However, it should be noted that CMR also has some limitations, including ECG-gated image acquisition, dependency on breath holding, lower image quality with arrhythmias, incompatibility with certain implants, a long acquisition time, and unsuitability for patients with claustrophobia [9].

Cardiac magnetic resonance is the standard imaging modality for functional quantification of right heart tumors. It is an imaging technique with a high spatial and temporal resolution. It provides an excellent depiction of the anatomy, contrast resolution, and tissue characterization; a wide field of view; multiplane acquisition; and reconstruction possibilities. Nevertheless, cardiac magnetic resonance has limited accessibility and requires higher expertise and a longer scan time. It is relatively expensive and not possible in patients with claustrophobia (unless intravenous anesthesia is administered), CMR-incompatible devices, or severe renal dysfunction, and it requires stable rhythm (this examination can be performed in hemodynamically stable patients). Even if rare, the risk of gadolinium deposition and nephrogenic systemic fibrosis exists. As for echocardiography and CT, right heart tumors could be an incidental finding on CMR; it allows for more accurate tissue characterization and tumor assessment, achieved with late gadolinium enhancement (LGE) sequences.

To date, CMR is considered the gold standard for volumes and myocardial mass assessment, especially of the right ventricle (i.e., cine imaging). It is highly appropriate for flow and shunt quantification, allowing for the hemodynamic assessment of valvular pathology (i.e., phase-contrast sequences) [3].

Tissue characterization is a major strength of CMR, achieved using T1- and T2-weighted, perfusion, and early and late gadolinium-enhanced (with normal and long inversion times) sequences, as well as with parametric mapping techniques such as T1, T2, and T2 mapping. A right ventricle thrombus typically has low signal intensity with all sequences, including late gadolinium-enhanced sequences at long inversion time, whereas a neoplasm shows variable signal intensity and contrast enhancement.

Prospective electrocardiographic triggering is adequate for morphologic evaluation, but retrospective electrocardiographic gating and the acquisition of data throughout the cardiac cycle are essential for dynamic and functional information. Right heart tumors require triphasic injection (e.g., contrast material injection followed by either a contrast material–saline mixture administered at the same flow rate or contrast material only administered at a slower flow rate than that of the first phase, followed by a saline bolus).

In addition, CMR can precisely delineate intra- and extracardiac anatomy through several sequences.

### 2.5. Positron Emission Tomography-Computed Tomography

The main strengths of PET–CT in assessing metabolic activity are staging malignant tumors, optimizing biopsy sites, guiding therapeutic management by allowing radiotherapy planning, and evaluating prognosis. Positron emission tomography has 100% sensitivity and 92% specificity for differentiating benign and malignant cardiac tumors [7]. However, this imaging method does not have high spatial and temporal resolutions, exposes patients to radiation, and requires a specific diet [9].

As an imaging method, PET–CT could offer the following intensity- and volume-based PET parameters of the cardiac mass [9]:

1. Maximum standardized uptake value;
2. Mean standardized uptake value;
3. Metabolic tumor volume;
4. Total lesion glycolysis.

These parameters enable differentiation between benign and malignant tumors since the mean standardized uptake value, metabolic tumor volume, and total lesion glycolysis are significantly higher in malignant lesions compared to benign ones [16].

PET–CT plays a crucial role in the early detection of occult or distant metastases. It proves valuable both before and after surgical resections, as it can unveil local metabolic activity persisting post excision in malignant tumors [17]. Therefore, PET–CT has a prognostic role in both short- and long-term follow-up.

In the case of rare malignancies like neuroendocrine tumors and cardiac paragangliomas, it is useful to perform molecular imaging (with 68Ga–PET DOTA(0)-Tyr(3)-octreotate) in order to better visualize and characterize these tumors.

PET–CT and CMR can independently diagnose benign and malignant lesions. CMR has higher sensitivity compared with FDG-PET–CT in distinguishing benign from malignant cardiac tumors, while FDG–PET–CT has higher specificity. The combination of these modalities increases the diagnostic yield for malignant tumors [18]. In patients with hybrid magnetic resonance/PET scanning, when it is desired to differentiate between benign and malignant cardiac masses, 18F-FDG–PET scanning has a sensitivity of 100% and a specificity of 92% [4]. However, these imaging tools should be used in specific clinical settings, such as the involvement of the great vessels or for disease-staging purposes [19].

The integration of positron emission tomography with CT or CMR in a hybrid approach improves the detection of malignant tumors. For example, a hybrid CMR-18F-fluorodeoxyglucose PET can identify cardiac metastatic melanoma [20]. 18F-FDG PET–CT is useful in distinguishing malignant from benign cardiac tumors when CT signs are inconclusive [16]. In the context of neuroendocrine tumors and cardiac paragangliomas, 68Ga–PET DOTA (0)-Tyr (3)-octreotate is recommended for enhanced visualization and characterization through molecular imaging [21].

### 2.6. Invasive Angiography

Invasive angiography is rarely used for the diagnosis of right heart tumors; it is merely reserved for specific cases (such as assessing the vascularization of tumors localized on the tricuspid or pulmonary valve). This procedure has excellent spatial and temporal resolution, provides a hemodynamic assessment of the tumors, and allows therapeutic interventions at the same time. However, being an invasive procedure, it could be associated with the following risks: bleeding, pseudo-aneurysm, infection, stroke, arrhythmia, and allergic reactions.

## 3. Imaging Features of Right Heart Tumors

Secondary cardiac tumors occur up to 40-fold more frequently than primary cardiac tumors [7], which are exceedingly rare in clinical practice. Most frequently, secondary cardiac tumors involve the right heart.

Benign cardiac tumors may be associated with malignant arrhythmias, embolism, or impaired hemodynamics, leading to life-threatening events, even if, from a histological point of view, these tumors have a good prognosis. However, patient history and clinical context, together with appropriate and early diagnosis of these tumors, are recommended. The use of multimodality imaging has improved diagnosis and management by enabling early detection and diagnosis, followed by timely and effective treatment.

**Figure 5:**
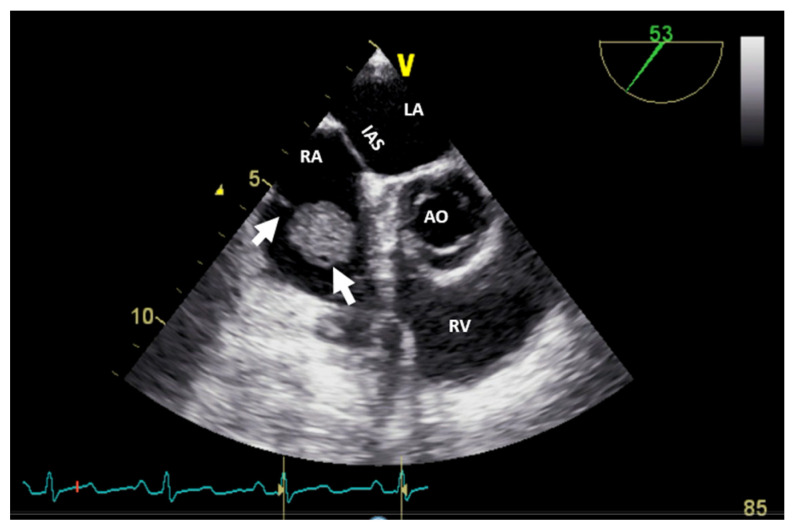
Two-dimensional transesophageal echocardiography (short-axis view at the level of the great vessels) showing a mass (arrow) on the port-a-cath in a patient with neoplasia and chemotherapy. It was an incidental finding. The mass disappeared after initiating anticoagulation treatment. AO, aorta; IVS, interventricular septum; LA, left atrium; LV, left ventricle; RV, right ventricle.

**Figure 6:**
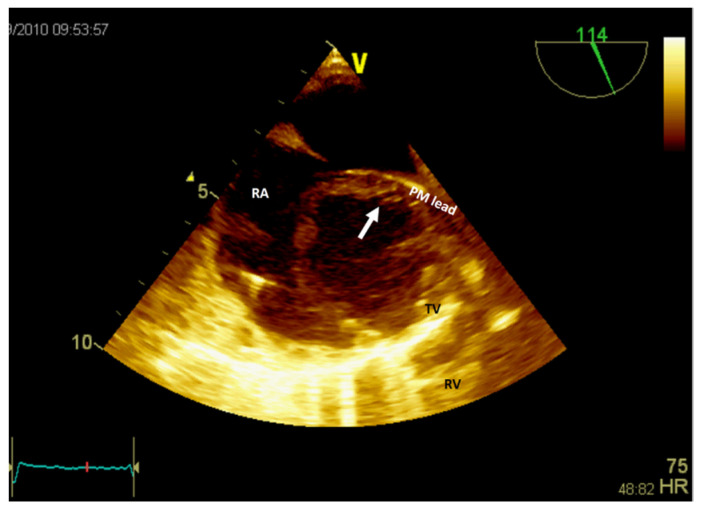
Two-dimensional transesophageal echocardiography (short-axis view at the level of the great vessels) showing a mass (arrow) in the right atrium on the pacemaker leads. PM, pacemaker; RA, right atrium; RV, right ventricle; TV, tricuspid valve.

The diagnostic approach for right heart tumors is primarily based on differentiating them from other right heart masses such as thrombi (Figure 5) or vegetations (Figure 6). For example, in a patient with neoplasia and a port-a-cath (whether undergoing chemotherapy or not), a right heart mass discovered incidentally on TTE or TEE suggests a thrombus on the port-a-cath (Figure 5). It is less likely to be a benign tumor, especially considering the fact that these patients undergo repeated TTE evaluations. The initiation of anticoagulant treatment can lead to the resolution of this mass. However, a differential diagnosis is mandatory, involving considerations of marantic endocarditis or metastases.

If the patient has a pacemaker or automatic implantable defibrillator, the first supposition in a clinical context suggestive of endocarditis is vegetation (Figure 6, Video S3—Supplementary Materials). A differential diagnosis must include fibrin formations or thrombi. However, if the patient is already anticoagulated (due to atrial fibrillation), these diagnoses are less likely.

Secondly, using TTE and/or TEE, the histology-based likelihood of the following characteristics is important: the tumor location and the morphological and functional characteristics [8].

The clinical presentations and main findings provided by imaging methods for the most frequently encountered benign right heart tumors are presented in Table 2 [6]. Echocardiography, CMR, cardiac CT, and PET all have complementary roles, requiring two or more of these imaging methods for an accurate diagnosis [6].

Almost half of primary benign cardiac tumors are **myxomas** (Figure 2, Table 2). Cardiac myxomas can arise in 20% of cases in the right atrium and around 3–4% in the right and left ventricles [6]. Up to 90% of myxomas are solitary and sporadic, with less than 10% being multiple and familial (Carney complex).

**Papillary fibroelastomas** (Video S4—Supplementary Materials) are the second most common benign cardiac tumors; they seem to surpass myxomas, representing about 10% of all cardiac tumors, and are frequently diagnosed in men between 40 and 80 years old [6]. These are usually located on cardiac valves (about 75% of all cardiac valvular tumors) and less often in the right heart [22]. In the presence of symptoms (such as pulmonary embolism on the right heart or transient ischemic attack on the left heart) or significant valvular regurgitation, surgical excision ensures an accurate diagnosis.

**Figure 7:**
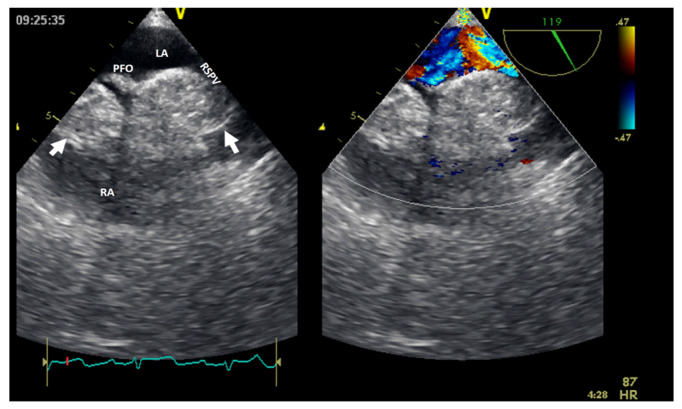
Two−dimensional transesophageal echocardiography with color Doppler (high transesophageal view at 119°) showing two tumors (arrow) at the level of the interatrial septum with compression on the right superior pulmonary vein (right image). LA, left atrium; LV, left ventricle; PFO, permeable foramen oval; RA, right atrium; RSPV, right superior pulmonary vein.

Cardiac **lipoma**, one of the right heart tumor types, is a very rare primary tumor without evidence of malignant transformation. Among benign cardiac tumors, lipomas seem to have an incidence between 2.9 and 8% [4]; they could be within the cardiac chamber (about 53%), encompassing all three layers of the cardiac wall (the endocardium, the myocardium in almost 11% of cases, or the pericardium in about 32% of cases) [8]. Cardiac lipomas within the cardiac chamber are usually smaller than those within the pericardium. Multimodal non-invasive imaging is important in the diagnosis (especially CMR), follow-up, and management of these tumors. There are many case reports in the literature of right atrial lipomas and fewer with right ventricle lipomas [8]; more than 90% of these patients underwent surgical resection [4,9]. However, this could fail if there is infiltrative growth into the myocardium. The confirmation of cardiac lipomas by anatomopathological assessment is mandatory. Lipomatous hyperplasia and hypertrophy of the myocardium interatrial septum, which are not considered true cardiac lipomas, could be obstacles to the transseptal puncture in patients undergoing an ablation procedure (Figure 7). Cardiac magnetic resonance can facilitate a differential diagnosis of liposarcoma before resection (if necessary).

The main difference is liposarcoma, which exhibits the following characteristics on CMR: prominent areas of enhancement associated with non-adipose lesions, thick or nodular septa, and prominent foci of high T2 signal [20]. The size and margins are also discriminatory signs between lipomas and liposarcomas. The latter is usually larger (>10 cm), with nodular margins and a lower fat content (<75%) [23]. Even with these imaging characteristics, without radiomics, well-differentiated liposarcomas might prove challenging to differentiate from mature cardiac lipomas.

**Rhabdomyomas** are more frequently diagnosed in infants and children, with an equal sex distribution. These can be either intramyocardial or intracavitary, situated in the left or right heart [6]. Frequently, they can be multiple, with intraluminal extensions in more than half of cases. In adults, suspicion of a malignant tumor (rhabdomyosarcoma, Video S5—Supplementary Materials) requires the extension of imaging explorations to CMR +/− PET scans.

**Fibromas** are tumors rarely diagnosed in adults, being the second most frequently occurring primary benign tumors in children, usually in the ventricles. These appear as non-capsulated masses located most commonly in the left-ventricle free wall (in over half of cases), followed by the right-ventricle free wall (almost one-third of cases) [6]. Cardiac fibromas have a poor prognosis.

In myxofibrosarcoma, a very rare malignant cardiac tumor with a poor prognosis, multimodality imaging is essential for appropriate diagnosis, optimal surgical planning, and the assessment of the response to oncological treatment during follow-up [7].

**Paraganglioma**, a rare neuroendocrine tumor, is typically diagnosed in young female adults (between 20 and 60 years old) and is infrequently localized in the heart [24]. There are no cases described in the right heart. However, up to 10% of paragangliomas are malignant [25]. Clinicians and imaging specialists can exclude this diagnosis only by echocardiography when a right ventricular mass is discovered (Table 2).

**Hemangiomas** are vascular tumors more frequently found in women, more commonly in the right atrium (Table 2). In these benign tumors, histological examination can establish a differential diagnosis between the various types of hemangiomas (Video S2—Supplementary Materials).

Primary malignant tumors, either in children or in adults, are mostly represented by sarcomas [8]. Angiosarcomas (most commonly in adults and the most frequent primary malignant tumor) and lymphomas can be identified in the right atrium and involve all layers of the heart [8]. Angiosarcomas are usually located near the inferior vena cava or involve the entire right atrial free wall, atrial roof, and interatrial septum. These can often be extended to the pericardium and to the right ventricle. Tumors identified in the pulmonary artery are usually intimal sarcomas; in right cardiac cavities, these can be spindle-cell sarcomas or leiomyosarcomas [8]. Inflammatory myofibroblastic tumors, teratomas, and yolk sac tumors are typical of fetuses and children; paragangliomas are more common in adults. Besides imaging modalities, clinical judgment and histologic examination play an essential role in the diagnostic management of these tumors.

## 4. Proposed Algorithm for an Appropriate Multimodality Imaging Diagnosis in Right Heart Tumors

Nowadays, there is enough information regarding histological findings and outcomes that allows for the formulation of standardized recommendations in multimodality imaging for the assessment of right heart tumors [26].

**Figure 8:**
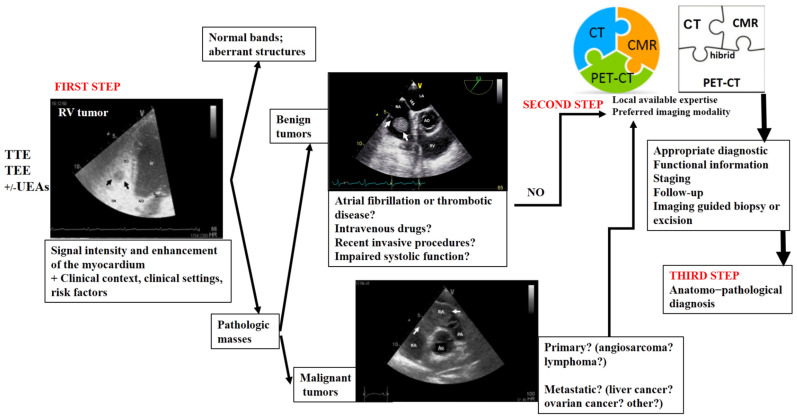
Multimodality imaging diagnostic algorithm for right heart tumors. TTE, transthoracic echocardiography; TEE, transesophageal echocardiography; UEAs, ultrasound-enhancing agents; CT, computer tomography; CMR, cardiac magnetic resonance; PET–CT, positron emission tomography-computer tomography.

In Figure 8, we propose an algorithm for the appropriate multimodality imaging diagnosis of right heart tumors. This proposed algorithm could serve as a practical guide not only for imaging specialists but also for clinicians who encounter a right heart mass. In many countries, echocardiography is performed by clinicians with expertise in this type of imaging modality. This can pose an important challenge in clinical practice, which requires extensive expertise. This algorithm is based on the aforementioned four objectives [4], along with the advantages and drawbacks of each imaging modality used in right heart tumors, as well as the features of these tumors.

A right heart tumor can be identified during a routine examination by transthoracic echocardiography or due to cardiac symptoms. The clinicians have useful tools to facilitate the evaluation of these masses. The first-line choice is assessment by echocardiography, with its multiple techniques. The obtained data must be interpreted in a clinical and biological context. The first three objectives of an ideal algorithm can be fulfilled by this first step of the proposed algorithm in many right ventricular masses.

The second step is represented by one, sometimes even two, of the more complex modality imaging methods. This choice is motivated not only by the advantages of each imaging modality but also by the local expertise or the preferred imaging modality in the center. The second and fourth objectives of the ideal algorithm can be more refined by this step of the proposed algorithm.

This step is followed by staging, follow-up, and/or imaging-guided excision or biopsy, which is usually performed in selected cases in order to obtain anatomopathological confirmation. This step of the proposed algorithm fulfills the third and fourth objectives. A transvenous biopsy is essential if there are features suggestive of malignancy or causing hemodynamic impairment. It should be performed even before the CT scan, MRI, or PET–CT (which are still relevant in the staging process), as this allows the timely initiation of chemotherapy. However, using a structured imaging approach, it is possible to reach an appropriate diagnosis without a biopsy.

Artificial intelligence in cardiovascular imaging has proven useful in areas such as detection, characterization, and monitoring of intracardiac masses, as well as image interpretation [27,28]. In the future, artificial intelligence is likely to facilitate the development of an algorithm for a practical and appropriate imaging diagnosis of right heart masses. Moreover, there is potential for artificial intelligence to integrate all medical data, including imaging, in order to predict the prognosis [27]. Nevertheless, practical approaches for multimodality cardiovascular imaging in cardio-oncology patients are gaining more and more traction [29].

This algorithm can be useful not only for imaging diagnosis but also for optimal therapeutic planning for any heart tumor. A multimodal, integrated imaging approach is crucial for an appropriate imaging diagnosis. A multidisciplinary team (including cardio-oncologists, cardiac surgeons, imaging specialists, hematologists, and sarcoma oncologists) plays an essential role in establishing the diagnostic and therapeutic management of malignant heart masses.

## 5. Conclusions

Right heart cardiac masses may be physiological (aberrant variants) or pathological structures. These are encountered either during a routine examination by transthoracic echocardiography or due to cardiac symptoms. Various imaging methods, following a multimodality imaging algorithm that concludes with the excision or biopsy of the tumor, are useful in diagnosis, therapy, and prognosis. In conclusion, in right heart tumors, there is no one-size-fits-all approach, as imaging methods often play complementary roles. Therefore, an integrated imaging approach is recommended.

## Figures and Tables

**Table 1:** The utility of the main imaging methods in right heart tumors.

IMAGING MODALITY	Identify, Localize, and Characterize the Tumor	Aid in the Differential Diagnosis by Distinguishing Them from Other Cardiac Pathologies	Evaluate the Consequences in the Case of Pathologic Entities	Classify as Primary or Secondary Tumor
2D/3D TTE/TEE (including UEAS)	++	++	++	+
CT (including contrast CT)	+++	+++	+++	++
CMR	+++	+++	+++	+++
PET	++	++	+	++++
Invasive angiography	NA	NA	+	NA
Nuclear imaging	NA	NA	NA	+++

Abbreviations: 2D/3D, two- or three-dimensional; CMR, cardiac magnetic resonance; CT, computer tomography; PET, positron emission tomography; TTE, transthoracic echocardiography; TEE, transesophageal echocardiography; UEA, ultrasound-enhancing agents. Note: Utility: NA—not applicable; + fair; ++ good; +++ very good; ++++ very, very good.

**Table 2:** Clinical presentations and imaging findings of benign right heart cardiac tumors.

Tumors. TUMORS TYPE AND CLINICAL PRESENTATIONS	ECHOCARDIOGRAPHY	COMPUTER TOMOGRAPHY	CARDIAC MAGNETIC RESONANCE	POSITRON EMISSION TOMOGRAPHY
MIXOMA: This is frequently diagnosed in middle-aged patients (30–60 years old). The typical triad of symptoms includes embolism, obstruction, and constitutional symptoms.	It is a mobile, round- or oval-shaped, heterogeneous echogenic mass attached to the endocardial surface, less commonly localized in the right atrium (especially in the left atrium, to the fossa ovalis). By contrast echocardiography, it appears as a partial or incomplete enhancement.	It appears as a heterogeneous, well-defined spherical or ovoid mass; a low-attenuation intracavitary mass with a lobular contour and calcification (which are more common in the right atrium); or as filling defects surrounded by enhanced intracardiac blood, hypo- or isoattenuating relative to the myocardium. Contrast-enhanced CT will show weak or absent enhancement.	Usually smooth, well-defined, lobular or oval mass; it appears heterogeneous or isointense on T1WI and heterogeneous or hyperintense on T2WI (because of the high extracellular water content). It is relatively hyperintense compared with the myocardium and hypointense relative to the blood pool. On resting: slight heterogeneous enhancement. On LGE images: patchy and more heterogeneous enhancement 10–15 min after gadolinium contrast administration.	It may manifest as a mildly hypermetabolic, hypodense area.
PAPILLARY FIBROELASTOMA: This can be an incidental finding or be associated with a cerebral or systemic embolic event.	It is small (usually less than 1.5 cm) and round; well-circumscribed; homogeneously textured appearance; a short pedicle; shimmering edges. It could be better visualized on 3D TEE, with echocardiography being the first step in the evaluation of embolic events.	It can be hard to see on moving valves. It appears as a focal low-attenuation mass with irregular borders on the valves’ surfaces. It can identify the anatomic location and attachment site, allowing simultaneous evaluation of the coronary arteries.	It can be hard to see on moving valves. It appears as a round, small, homogeneous mass attached to valvular leaflets. It exhibits isointense signal intensity relative to the myocardium on T1-weighted images and hypointense or hyperintense signal intensity on T2-weighted images. On cine CMR images, there is a hypointense signal intensity. There is usually no delayed gadolinium enhancement on LGE.	It is usually not necessary.
LIPOMA: This is frequently an incidental finding. It may be asymptomatic (even in large dimensions); fatigue; dyspnea on exertion; chest distress; palpitations; sudden death.	It identifies lipoma location and attachment, shape, and size. It appears to have hypoechogenic, homogenous echo intensity and well-defined borders.	Homogenous, encapsulated hypodense mass (between –45 HU and −100 HU).	It is essential in the differential diagnosis of liposarcomas. It has the same signal intensity as subcutaneous fat; hyperintense (T1W1 and T2W1); hypointense (in fat saturation sequences); T1/T2 value (ms): 255/65; post gadolinium: no enhancement.	It is usually not necessary, except in cases of suspected malignancy.
RHABDOMYOMA: This may be associated with symptoms of congestive heart failure, palpitations, or syncope. These symptoms may gradually disappear because of the spontaneous regression of the tumor.	It appears small, round, and lobulated.	Small, round, multiple homogenous hyperechoic masses of variable size, usually brighter than the surrounding myocardium; in contrast CT, it is hypodense. This allows differentiation from fibromas by deformation imaging.	On T1W1, rhabdomyomas appear isointense to slightly hyperintense (as on T2W1) compared with the surrounding myocardium. No delayed gadolinium enhancement on LGE.	It is usually not recommended, except in cases of suspected malignancy.
FIBROMA: This may be associated with fatal arrhythmias, heart failure, and sudden death. Surgical treatment is recommended, regardless of symptoms.	It appears as a large, intramural, well-delimited, non-contractile, solitary solid lesion within the myocardium, with central calcification.	It is described as an intramural, homogenous structure with sharp margins or infiltrative, with central calcification (a common feature of fibromas on CT), and soft-tissue attenuation, frequently without enhancement.	On T1WI, it is an isointense tumor, and on T2WI, it is a hypointense homogenous structure. On LGE, an intense delayed hyperenhancement is observed, without enhancement on resting.	It is usually not necessary, except in cases of suspected malignancy.
PARAGANGLIOMA: It is associated with symptoms such as tachycardia, tremors, palpitations, flushing, hypertension, or hypotension because of excessive secretion of catecholamines.	It appears as a granular, oval, heterogeneous structure, well-delimited, with a broad base. Sometimes, adjacent structures, like the superior vena cava, can be compressed. They are highly vascular structures.	It is a well-delimited heterogeneous structure with low attenuation. In contrast CT, it is characterized by marked heterogeneous enhancement. If the margins are poorly defined, invasion or extracardiac extension can be suspected. Coronary angiography is able to assess the relationship with the tumor.	It appears on T1WI as isointense or hypointense, and on T2WI as hyperintense, heterogeneous, and with peripheral rim enhancement.	It appears positive with an intense uptake of radiotracers.
HEMANGIOMA: It is an incidental finding; if symptomatic, the patient can present with chest pain, arrhythmias, heart failure, dyspnea on exertion, syncope, stroke, pericardial effusion, cardiac tamponade, and even sudden death.	It appears as a well-defined structure, endocardial or intramural, with oscillations during the cardiac cycle, good vascularization (it presents blood flow signals on color Doppler flow imaging), and obvious enhancement.	It is a well-defined structure with low or equal density, associated with heterogeneous intense enhancement and “vascular blush” on coronary angiography.	It appears on T1WI as heterogeneous isointense or hypointense, and on T2WI as hyperintense with heterogeneous enhancement.	It is usually not necessary, except in cases of suspected malignancy.

CMR, cardiac magnetic resonance; CT, computer tomography; HU, Hounsfield units; LGE, late gadolinium enhancement; T1W1, T1-weighted image; T2W1, T2-weighted image.

## Data Availability

Not applicable.

## Supplementary Materials

The following supporting information can be downloaded at: https://www.mdpi.com/article/10.3390/jcm13041000/s1. Video S1: Two-dimensional transthoracic echocardiography (subcostal view) showing the presence of a thick right ventricular free wall (which on histological diagnosis was a myosarcoma); Video S2: Contrast transthoracic echocardiography image with agitated saline serum (two-dimensional echocardiography in apical 4-chamber view) showed a spherical, mobile tumor in the right ventricle area, attached to the tricuspid valve (which on histological diagnosis was a cavernous hemangioma). Video S3: Two-dimensional transesophageal echocardiography (short-axis view at the level of the great vessels) showing a mass in the right atrium on the pacemaker leads. Video S4: Two-dimensional transesophageal echocardiography (short-axis view at the level of the great vessels with focus on the tricuspid valve and the right ventricle) showing a mass in the right ventricle, which was a papillary fibroelastoma on histological exam. Video S5: Two-dimensional transesophageal echocardiography (short-axis view at the level of the great vessels) showing a mass in the right ventricle (rhabdomyosarcoma).
